# Administering an appeasing substance to optimize welfare and performance of receiving cattle^1^

**DOI:** 10.1093/tas/txaa086

**Published:** 2020-12-22

**Authors:** Eduardo A Colombo, Reinaldo F Cooke, Alice P Brandão, Jacob B Wiegand, Kelsey M Schubach, Consuelo Sowers, Glenn C Duff, Vinicius N Gouvêa, Bruno I Cappellozza

**Affiliations:** 1 Department of Animal Science, Texas A&M University, College Station, TX; 2 Clayton Livestock Research Center, New Mexico State University, Clayton, NM; 3 Nutricorp, Araras, Brazil

## INTRODUCTION

Feedlot receiving is one of the most critical phases within the beef production cycle, when cattle are exposed to a multitude of stress and health challenges that directly impact animal welfare and productivity (Duff and Galyean, 2007). These include road transport, commingling with different animals, and exposure to novel diets and environments (Cooke, 2017), which elicit adrenocortical and acute-phase protein responses known to impair cattle immunocompetence and growth (Carroll and Forsberg, 2007). Accordingly, incidence of bovine respiratory diseases (BRDs) is extremely elevated during feedlot receiving, with clinical symptoms observed in up to 60% of receiving cattle (Snowder et al., 2006).

With increased restrictions regarding the use of feed-grade antimicrobials in livestock systems, management strategies to minimize stress and enhance cattle performance and immunity are warranted. One example includes the use of the bovine appeasing substance (BAS); a mixture of fatty acids that replicate the composition of the original bovine appeasing pheromone (Osella et al., 2018). Recent research from our group reported that BAS administration to beef calves at weaning, and to beef bulls upon feedlot arrival improved initial body weight (BW) gain (Cooke et al., 2020). However, research investigating the effects of BAS administration in cattle is still limited, and the biological mechanisms behind the aforementioned results warrant further investigation. We hypothesized that administration of BAS to beef cattle upon feedlot arrival will alleviate adrenocortical and acute-phase protein responses, improve feed intake and efficiency, resulting in improved performance during a 45-d receiving period. Hence, this study evaluated the impacts of BAS administration at feedlot arrival on BW gain, BRD incidence, and physiological responses of receiving cattle.

## MATERIALS AND METHODS

This experiment was conducted at the New Mexico State University—Clayton Livestock Research Center (Clayton, NM). All animals were cared for in accordance with acceptable practices and experimental protocols reviewed and approved by the New Mexico State University—Institutional Animal Care and Use Committee.

### Animals and Treatments

Three hundred and forty-two recently weaned Angus-influenced steers were purchased from a commercial auction facility (Cattlemen’s Livestock Commission Company, Dalhart, TX). Steers were originated from 16 cow–calf operations located in northern TX. On the day of purchase (d −1; 1800 h), steers were loaded into four commercial livestock trailers (Legend 50’ cattle liner; Barrett LLC, Purcell, OK) at the auction yard and transported for 12 h to stimulate the stress of a long-haul (Cooke, 2017). On d 0 of the experiment, steers were unloaded at the Clayton Livestock Research Center and arrival BW was recorded. Steers were ranked by source and shrunk BW, assigned to receive BAS (Nutricorp; Araras, Brazil; *n* = 171) or placebo (diethylene glycol monoethyl ether; CON; *n* = 171), and immediately segregated by treatment into one of two groups and processed again for treatment administration. Treatments (5 mL) were applied topically to the nuchal skin area of each animal (Cooke et al., 2020). Treatment groups were maintained in two separate paddocks with free-choice hay, water, and mineral supplement for 24 h, with an empty paddock between groups to maintain distance.

On d 1 of the experiment, steers within treatment groups were ranked by source and shrunk BW and allocated to a 24 pen drylot (35 × 12 m; 14 to 15 steers/pen; 12 pens/treatment group), in a manner that pens had equivalent initial shrunk BW and steers from at least 4 sources to stimulate the stress of commingling. Steers had free-choice access to water and RAMP (Cargill, Dalhart, TX), which was offered twice daily (0800 and 1300 h) from d 0 to 45 in a manner to yield 10% residual orts. Pens differing in treatment were not adjacent to each other. Steers were vaccinated and administered anthelmintic on d 0 (Lopez et al., 2018).

### Sampling and Laboratorial Analyses

Steer BW was recorded on d 1, 7, 17, 31, and 45, whereas individual average daily gain (ADG) calculated by modeling linear regression of BW against sampling days. Feed intake (dry matter basis) from each pen was evaluated daily, divided by the number of steers within each pen and expressed as kg per steers/d. Total BW gain and feed intake of each pen were used for feed efficiency (G:F) calculations. Steers were observed daily for BRD signs according to the DART system (Zoetis, Florham Park, NJ) and received antimicrobial treatment as in Lopez et al. (2018). Animals were removed from the experiment if a third medical treatment was warranted.

Five animals were randomly selected within each pen on d 0 and assigned to collection of blood samples concurrently with BW evaluations. Blood was collected into commercial blood collection tubes (Vacutainer, 10 mL; Becton Dickinson, Franklin Lakes, NJ) containing freeze-dried sodium heparin for plasma collection. All blood samples were placed immediately on ice, centrifuged (2,500 × g for 30 min; 4 °C) for plasma harvest and stored at −80 °C on the same day of collection. Plasma samples were analyzed for haptoglobin and cortisol as described by Cooke et al. (2020), with intra and interassay coefficients of variation ≤10%.

### Statistical Analysis

Steer was considered the experimental unit for all analyses. Quantitative data were analyzed using the MIXED procedure of SAS (SAS Inst. Inc., Cary, NC), whereas binary data were analyzed using the GLIMMIX procedure of SAS (SAS Inst. Inc.). All data were analyzed with steer(pen × treatment) and pen(treatment), but for feed intake and G:F that used pen(treatment) as the random variable. Model statements contained the effects of treatment in addition to day and the resultant interaction for repeated measures. Plasma variables were analyzed using results from d 0 as covariate. The specified term for all repeated statements was day, with pen(treatment) as subject for feed intake and steer(pen × treatment) as subject for all other analyses. The covariance structure used was first-order autoregressive, which provided the smallest Akaike information criterion. Results are reported as least square means, or covariately adjusted least square means for plasma variables, and separated using least square differences. Significance was set at *P* ≤ 0.05 and tendencies at *P* > 0.05 and ≤ 0.10. Results are reported according to main effects if no interactions were significant, or according to the highest order interaction detected.

## RESULTS

As designed, initial BW (d 0) was similar (*P* = 0.97) between treatments (Table 1). Average daily gain was greater (*P* = 0.05) in BAS vs. CON steers, although final BW did not differ (*P* = 0.36) between treatments (Table 1). No treatment effects were detected for feed intake (*P* = 0.95), resulting in greater (*P* = 0.05) G:F in BAS vs. CON steers (Table 1).

**Table 1.** Performance parameters of beef steers receiving (BAS; *n* = 171) or not (CON; *n* = 171) a BAS at feedlot entry (d 0)^*a*^

Item	CON	BAS	SEM	*P*-value
Body weight, kg				
d 0	262	261	3	0.97
d 7	243	243	3	0.99
d 17	254	257	3	0.42
d 31	267	273	3	0.15
d 45	291	295	3	0.36
Average daily gain, kg/d	0.85	1.00	0.05	0.05
Feed intake, kg/d	4.95	4.98	0.21	0.95
Feed efficiency, g/kg	142	171	10	0.05

^*a*^Feed intake was recorded daily from d 1 to 45 by measuring offer and refusals from each pen, divided by the number of steers within each pen, and expressed as kg per steer/d. Feed efficiency was calculated using total feed intake from d 1 to 45, and BW gain of each pen from d 1 to 45. Average daily gain calculated by modeling linear regression of BW against sampling days (1, 7, 17, 31, and 45).

**Figure 1.**
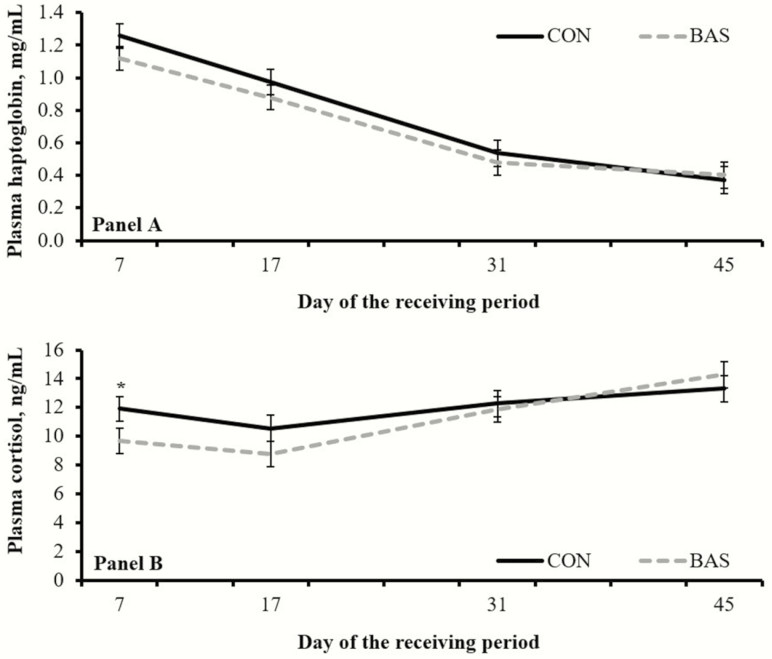
Concentrations of plasma haptoglobin (Panel A) and cortisol (Panel B) in beef steers receiving (BAS; *n* = 171) or not (CON; *n* = 171) a BAS at feedlot entry (d 0). Values from d 0 were used as independent covariate in each respective analysis. No treatment differences were noted (*P* ≥ 0.37) for plasma haptoglobin, whereas tendency for treatment × day interaction was detected (*P =* 0.07) for plasma cortisol. Within days: *P = 0.05.

**Figure 2.**
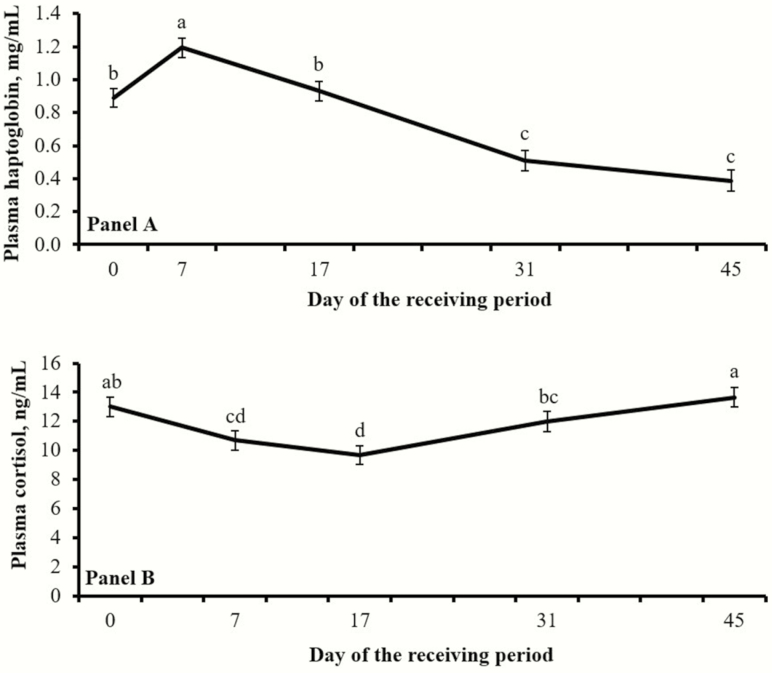
Concentrations of plasma haptoglobin (Panel A) and cortisol (Panel B) from beef steers relative to feedlot arrival (d 0). Day effects were detected (*P* < 0.01) for both variables. Days with different superscripts (a, b, c, d) differ (*P* ≤ 0.05).

No treatment effects were detected (*P* ≥ 0.37) for concentrations of plasma haptoglobin (Fig. 1). A tendency for a treatment × day interaction was noted (*P* = 0.07) for plasma cortisol concentrations, which were greater (*P* = 0.05) in CON vs. BAS steers on d 7 (Fig. 1). Day effects were detected for both plasma variables (Fig. 2).

A treatment × day interaction was detected (*P* < 0.01) for BRD incidence, which was greater (*P* ≤ 0.05) in BAS vs. CON steers on d 6 to 10 and d 18 to 21 (Fig. 2). However, no treatment effects were detected (*P* = 0.24) for overall incidence of BRD signs during the 45-d receiving period (Table 2). The number of antimicrobial treatments required per steer diagnosed with BRD symptoms to recover from sickness was greater (*P* = 0.04) in CON vs. BAS steers (Table 2). No treatment differences were detected (*P* ≥ 0.41) for mortality incidence, or proportion of steers removed from the experiment (Table 2).

**Table 2.** Morbidity parameters from beef steers receiving (BAS; *n* = 171) or not (CON; *n* = 171) a BAS at feedlot entry (d 0)a

Item	CON	BAS	SEM	*P*-value
Incidence of BRD signs, %	75.4	81.3	0.03	0.24
Number of antimicrobial treatments required	1.54	1.41	0.05	0.04
Steers removed from experiment, %	15.2	14.6	0.03	0.88
Mortality, %	12.2	8.8	0.03	0.41

^*a*^Steers were observed daily for BRD signs according to the DART system (Zoetis, Florham Park, NJ), and received antimicrobial treatment as in Lopez et al. (2018). Steers were removed from the study if a third medical treatment was warranted.

## DISCUSSION

**Figure 3.**
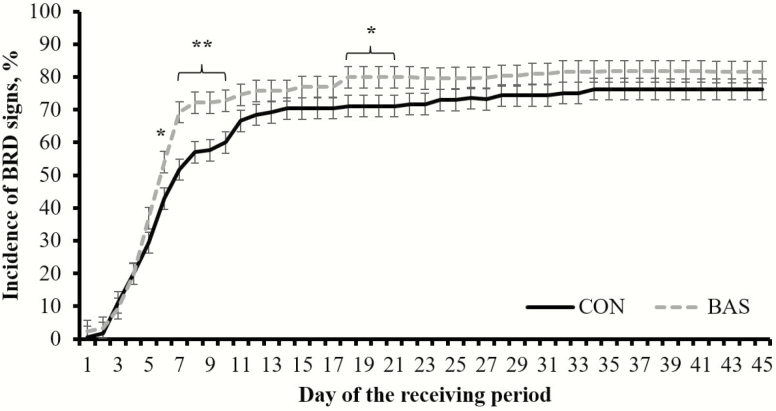
Cumulative incidence of BRD symptoms in beef steers receiving (BAS; *n* = 171) or not (CON; *n* = 171) a BAS at feedlot entry (d 0). Steers were observed daily for BRD signs according to the DART system (Zoetis, Florham Park, NJ) and received antimicrobial treatment as in Lopez et al. (2018). A treatment × day interaction was detected (*P* < 0.01). Within days: *P ≤ 0.05; **P < 0.01.

Steers utilized in this experiment were considered high risk, given their prior management and health history were not fully known (Wilson et al., 2017). Furthermore, steers experienced the stress of weaning, auction, transportation, commingling, vaccination, and feedlot entry within a 72-h period, the combination of which impacts cattle immunocompetence and performance (Cooke, 2017). Day effects observed for plasma cortisol and haptoglobin (Fig. 2) corroborate that steers experienced an adrenocortical and subsequent acute-phase protein response elicited by transportation and feedlot entry (Cooke, 2017). Collectively, this stress-induced inflammation is linked with the BRD complex in receiving cattle (Cooke, 2017) supporting the substantial incidence of BRD observed herein (Table 2; Fig. 3), which is comparable to research conducted in commercial receiving yards (Snowder et al., 2006).

Treatment differences in BRD incidence during the experimental period (Fig. 3) indicate BAS administration resulted in increased susceptibility to BRD. In turn, it can be speculated that BAS administration upon feedlot arrival resulted in earlier detection of illness compared with CON steers, and hence fewer antimicrobial treatments to recover from sickness (Table 2). Diagnosis of BRD requires behavioral evaluation to identify and treat, given changes in behavior such as decreased activity and abnormal feeding and drinking behavior are indicative of general malaise (Weary et al., 2009). Cattle are prey species that mask any signs of vulnerability, especially if the sickness makes them an easier target for predation. Therefore, many subclinical BRD cases are well disguised by cattle as part of their natural defense (Thompson et al., 2006). Results from this experiment may suggest that BAS administration upon feedlot entry exerted, at least partially, a calming effect and thereby enabled earlier detection of BRD and rapid treatment of disease.

Steer ADG during the receiving period was improved by BAS administration, which supports the aforementioned rationale given that ADG is negatively associated with BRD incidence (Wilson et al., 2017). Moreover, improved ADG in BAS steers should be primarily attributed to increased G:F feed efficiency, given that feed intake during the 45-d receiving period was similar between treatments (Table 1).

Previous research has suggested that BAS is active within the first 15 d upon administration (Osella et al., 2018; Cooke et al., 2020). Accordingly, plasma concentrations of cortisol were reduced in BAS steers on d 7, suggesting that BAS alleviated the adrenocortical response typical of the receiving period (Carroll and Forsberg, 2007). Elevated cortisol has been positively associated with circulating haptoglobin (Cooke, 2017), while BAS and CON steers had similar plasma concentrations of haptoglobin throughout the receiving period. Numerical differences in plasma haptoglobin concentrations noted herein, particularly on d 7, may also suggest that subsampling yielded a type II statistical error for this variable.

## IMPLICATIONS

This experimental model fully represented the stress and health challenges experienced by commercial cattle during feedlot receiving, resulting in substantial BRD incidence and morbidity. Administration of BAS upon feedlot entry improved feedlot receiving ADG by enhancing G:F. Administration of BAS facilitated earlier detection of BRD and reduced the need for antimicrobial treatments, which may also have contributed to improved performance responses. Collectively, these results suggest BAS administration as a promising strategy to benefit performance and immunocompetence of feedlot receiving cattle.
